# Draft Genome Sequences for Ten *Salmonella enterica* Serovar Typhimurium Phage Type 135 Variants

**DOI:** 10.1128/genomeA.00293-13

**Published:** 2013-06-06

**Authors:** Geoff Hogg, Karolina Dimovski, Lester Hiley, Kathryn E. Holt

**Affiliations:** Microbiological Diagnostic Unit Public Health Laboratory, Department of Microbiology and Immunology, University of Melbourne, Parkville, Victoria, Australiaa; Queensland Health, Archerfield, Queensland, Australiab; Department of Biochemistry and Molecular Biology, Bio21 Institute, University of Melbourne, Parkville, Victoria, Australiac

## Abstract

*Salmonella enterica* serovar Typhimurium (*S*. Typhimurium) is a common cause of gastroenteritis in humans. Here, we report the draft genome sequences of 10 isolates of an *S*. Typhimurium phage type 135 variant that is linked to egg-associated outbreaks in Tasmania, Australia.

## GENOME ANNOUNCEMENT

*Salmonella enterica* serovar Typhimurium (*S*. Typhimurium) is a common cause of gastroenteritis in humans (1, 2). The first *S*. Typhimurium genome was published in 2001 (3), and only a handful of additional *S*. Typhimurium genomes have been published since (4). In Australia, a variant of *S*. Typhimurium phage type 135 (sometimes referred to as 135a but without the official phage-type designation) is a common form of *S*. Typhimurium that is responsible for food-borne gastroenteritis (5, 6) and has been associated with multiple outbreaks (7, 8). To facilitate studies of *S*. Typhimurium 135a and future outbreak investigations, the genomes of 10 *S*. Typhimurium 135a isolates linked to egg-associated outbreaks in Tasmania were sequenced. These include five isolates from 2005, one from 2006, two from 2007, and three from 2008.

Sequencing was performed on Illumina HiSeq (10 isolates multiplexed in one lane), generating paired-end 100-bp reads. Reads were assembled using Velvet and Velvet Optimizer (9), resulting in a median of 240 contigs per genome (range, 210 to 284 contigs), covering a median of 4.69 Mbp of sequence (range, 4.64 to 4.73 Mbp), with N_50_ of 99 kbp to 250 kbp and a mean read depth of 300× to 1,000×.

Read mapping to the available finished *S*. Typhimurium reference sequences (10) revealed the closest reference for all isolates was *S*. Typhimurium SL1344 (phage type DT44; accession no. NC_016810.1). Each set of contigs was ordered against *S*. Typhimurium SL1344 using ABACAS (http://abacas.sourceforge.net/) and annotated using the NCBI Prokaryotic Genome Automated Annotation Pipeline (PGAAP). Prophage sequences were identified using PHAST (11).

Between 4,780 and 4,859 protein-coding genes were annotated in each genome, with the exception of STm2, which carries 4,943 genes due to the presence of an additional ~95 kbp of novel sequence with high similarity to the colicin plasmid PColIb-P9 (accession no. AB021078.1) and carries the structural and immunity genes of colicin Ib (12). Multiple alignment of the assemblies using Mauve (13) revealed that the novel *S*. Typhimurium genomes were nearly identical in DNA content, were identical in prophage content, and each carried a copy of the *S*. Typhimurium virulence plasmid pSLT (accession no. NC_003277 [3]). The novel *S*. Typhimurium genomes were very similar in gene content to that of DT44 strain SL1344, with just a few differences in prophage content. The Gifsy-2 and ST64B prophage sequences of SL1344 (SLP105, SLP203) were present and intact at the same locations within the *S*. Typhimurium phage type 135 genomes. The latter also contained a divergent copy of the Gifsy-1 prophage (SLP272) of SL1344 in the same location as in SL1344 and a novel 42.5-kb prophage sequence occupying the same insertion site as the SopEФ-P4 prophage sequences of SL1344 (SLP285 linked to SLP289). The novel phage consists of a 32-kbp P2 phage with 50.5% G+C content, linked directly to a 10.2-kbp P4 phage with 47.5% G+C content that is identical to SLP289 in *S*. Typhimurium SL1344. The P2 phage has no homology with SopEФ but shows significant levels of homology with phage sequences in *S. enterica* serovar Newport strain SL254 and *S. enterica* serovars Paratyphi A, Paratyphi C, and Heidelberg.

### Nucleotide sequence accession numbers. 

The annotated *S*. Typhimurium whole-genome sequences were deposited as Whole-Genome Shotgun projects at DDBJ/EMBL/GenBank under the accession numbers listed in Table 1.

**TABLE 1:** Accession numbers of the annotated *S*. Typhimurium whole-genome sequences

*S*. Typhimurium isolate	Accession no.	Version described here
STm1	AMDX00000000	AMDX01000000
STm2	AMDY00000000	AMDY01000000
STm3	AMEB00000000	AMEB01000000
STm4	AMEC00000000	AMEC01000000
STm6	AMED00000000	AMED01000000
STm8	AMDZ00000000	AMDZ01000000
STm9	AMEA00000000	AMEA01000000
STm10	AMEE00000000	AMEE01000000
STm11	AMEF00000000	AMEF01000000
STm12	AMEG00000000	AMEG01000000

## References

[1] Majowicz SE Musto J Scallan E Angulo FJ Kirk M O’Brien SJ Jones TF Fazil A Hoekstra RM, International Collaboration on Enteric Disease ‘Burden of Illness’ Studies The global burden of nontyphoidal salmonella gastroenteritis. Clin. Infect. Dis. 50:882–889 20158401 10.1086/650733

[2] Hendriksen RS Vieira AR Karlsmose S Lo Fo Wong DM Jensen AB Wegener HC Aarestrup FM 2011 Global monitoring of salmonella serovar distribution from the World Health Organization Global Foodborne Infections Network Country Data Bank: results of quality assured laboratories from 2001 to 2007. Foodborne Pathog. Dis. 8:887–900 21492021 10.1089/fpd.2010.0787

[3] McClelland M Sanderson KE Spieth J Clifton SW Latreille P Courtney L Porwollik S Ali J Dante M Du F Hou S Layman D Leonard S Nguyen C Scott K Holmes A Grewal N Mulvaney E Ryan E Sun H Florea L Miller W Stoneking T Nhan M Waterston R Wilson RK 2001 Complete genome sequence of *Salmonella enterica* serovar Typhimurium LT2. Nature 413:852–856 11677609 10.1038/35101614

[4] Pang S Octavia S Reeves PR Wang Q Gilbert GL Sintchenko V Lan R 2012 Genetic relationships of phage types and single nucleotide polymorphism typing of *Salmonella enterica* serovar Typhimurium. J. Clin. Microbiol. 50:727–734 22205813 10.1128/JCM.01284-11 PMC3295175

[5] Sintchenko V Wang Q Howard P Ha CW Kardamanidis K Musto J Gilbert GL 2012 Improving resolution of public health surveillance for human *Salmonella enterica* serovar Typhimurium infection: 3 years of prospective multiple-locus variable-number tandem-repeat analysis (MLVA). BMC Infect. Dis. 12:78 22462487 10.1186/1471-2334-12-78 PMC3368731

[6] OzFoodNet Working Group 2009 Monitoring the incidence and causes of diseases potentially transmitted by food in Australia: annual report of the OzFoodNet network, 2008. Commun. Dis. Intell. 33:389–413 10.33321/cdi.2009.33.42 20301968

[7] Stephens N Coleman D Shaw K 2008 Recurring outbreaks of *Salmonella* Typhimurium phage type 135 associated with the consumption of products containing raw egg in Tasmania. Commun. Dis. Intell. Q Rep. 32:466–468 19374277 10.33321/cdi.2008.32.47

[8] Stephens N Sault C Firestone SM Lightfoot D Bell C 2007 Large outbreaks of *Salmonella* Typhimurium phage type 135 infections associated with the consumption of products containing raw egg in Tasmania. Commun. Dis. Intell. Q Rep. 31:118–124 17503652 10.33321/cdi.2007.31.8

[9] Zerbino DR Birney E 2008 Velvet: algorithms for *de novo* short read assembly using de Bruijn graphs. Genome Res. 18:821–829 18349386 10.1101/gr.074492.107 PMC2336801

[10] Holt KE Baker S Weill FX Holmes EC Kitchen A Yu J Sangal V Brown DJ Coia JE Kim DW Choi SY Kim SH da Silveira WD Pickard DJ Farrar JJ Parkhill J Dougan G Thomson NR 2012 Shigella sonnei genome sequencing and phylogenetic analysis indicate recent global dissemination from Europe. Nat. Genet. 44:1056–1059 22863732 10.1038/ng.2369 PMC3442231

[11] Zhou Y Liang Y Lynch KH Dennis JJ Wishart DS 2011 PHAST: a fast phage search tool. Nucleic Acids Res. 39:W347–W352 21672955 10.1093/nar/gkr485 PMC3125810

[12] Mankovich JA Hsu CH Konisky J 1986 DNA and amino acid sequence analysis of structural and immunity genes of colicins Ia and Ib. J. Bacteriol. 168:228–236 3531169 10.1128/jb.168.1.228-236.1986 PMC213442

[13] Darling AE Mau B Perna NT 2010 progressiveMauve: multiple genome alignment with gene gain, loss and rearrangement. PLoS One 5:e11147 20593022 10.1371/journal.pone.0011147 PMC2892488

